# Beliefs affecting ART adherence in newly diagnosed HIV-positive participants in Manzini, Eswatini

**DOI:** 10.4102/sajhivmed.v25i1.1601

**Published:** 2024-09-25

**Authors:** Thabiso Mango, Mambwe Kasese-Hara, Mamakiri Mulaudzi

**Affiliations:** 1 Department of Psychology, Faculty of Humanities, School of Human and Community Development, University of the Witwatersrand, Johannesburg, South Africa

**Keywords:** HIV, antiretroviral therapy, adherence, PLHIV, Theory of Planned Behaviour, Eswatini

## Abstract

**Background:**

Achieving optimal adherence to antiretroviral therapy (ART) is challenging. Consistency in HIV care and treatment is crucial for achieving viral load suppression and preventing HIV-related illnesses, disease progression to AIDS, mortality, drug resistance, and onward transmission.

**Objectives:**

The purpose of this research was to gain a comprehensive understanding of the beliefs that play a role in determining the level of ART adherence among individuals newly diagnosed with HIV. By examining these beliefs, the researchers aimed to identify potential barriers and facilitators to adherence, ultimately contributing to the development of effective interventions and strategies to improve ART adherence.

**Method:**

An exploratory qualitative approach was employed in this study, utilising the Theory of Planned Behaviour (TPB) as its theoretical framework. To gather insights, in-depth interviews were conducted with 19 participants recruited post diagnosis, who shared their beliefs regarding ART adherence. Thematic analysis identified beliefs, categorised under TPB precursors, namely behavioural outcomes, subjective norms, and perceived behavioural control.

**Results:**

Participants emphasised health improvement, treatment effectiveness, and disease prevention as advantages to ART adherence, while disadvantages included fear of lifelong commitment, side effects, and stigma. ART adherence was enhanced by family support but impeded by a number of social factors. Participants expressed confidence in creating personal reminders or seeking external help, but anticipated various challenges.

**Conclusion:**

The research has shown that the beliefs affecting ART adherence in individuals recently diagnosed with HIV but not yet on treatment are like those that have been reported to influence adherence in HIV-positive participants currently receiving treatment.

**What this study adds:** This study provides insight into the beliefs held by individuals newly diagnosed with HIV regarding adherence to ART prior to initiating treatment.

## Introduction

Eswatini exhibits the highest prevalence of HIV globally, with about 19% of its population, or 26.8% of people aged 15 to 49 years, being infected with HIV.^1^ HIV or AIDS was the main cause of death in the country from the year 2000 to 2020.^2,3^ However, Eswatini has made progress in fighting the HIV epidemic, such that the HIV prevalence rate among people aged 15 years and above has dropped from 27.6% in 2010 and is estimated to be 24.4% by 2023.^3^

This improvement is largely due to the increased access to antiretroviral therapy (ART) across the country, as Eswatini has the highest rate of ART coverage for people living with HIV (PLHIV) in the sub-Saharan Africa region, with 91% of PLHIV receiving ART, compared to the regional average of only 78% of PLHIV on ART in 2021.^4^

Viral suppression is the main goal of HIV care and treatment, as it protects the health and increases the lifespan of the participant, and prevents the transmission of the virus to others.^5^ However, even with high ART coverage, only 73.1% of adults who test positive for HIV achieve viral suppression.^6^

One of the major barriers to viral suppression is poor adherence to medication which, according to the World Health Organization, ought to be at least 95% for optimal ART outcomes.^7,8,9^ The first year after a positive HIV test and starting treatment is a critical time for individuals to adjust to their new condition. Studies in Eswatini show that many people become unreachable within the first 6–12 months of ART, indicating the need to improve participant retention in HIV care.^10,11^

Poor participant retention can result in low adherence to ART, which negatively affects viral suppression and, consequently, participant health outcomes, and the low viral suppression rate of 73.1% in Eswatini suggests challenges in sustaining optimal adherence to HIV care and treatment, requiring specific interventions and strategies.

The literature extensively examines and documents the factors that influence adherence to ART among individuals who are already taking treatment. However, there is a significant research gap when it comes to studying the viewpoints of individuals who have recently been diagnosed with HIV but have not yet started ART. It is imperative to comprehend the factors and beliefs that play a role in determining the level of adherence to ART in this particular group in order to develop efficacious interventions that facilitate the initiation and continuation of their treatment.

### Conceptual framework

The research is based on Ajzen’s Theory of Planned Behaviour (TPB), which predicts intentional health behaviours.^12,13,14^ The TPB uses an elicitation qualitative study to identify specific beliefs held by the population being studied towards a particular behaviour. This method helps identify the core beliefs needed to initiate change in a specific population (newly diagnosed HIV-positive individuals) and environment (Manzini, Eswatini).^15^ Beliefs are considered to be a fundamental component of the TPB, as they are believed to form the cognitive and emotional basis for attitudes, subjective norms, and perceptions of behaviour. Specifically, behavioural beliefs influence the development of a positive or negative attitude towards a particular behaviour; normative beliefs lead to the perception of social pressure or subjective norms; and control beliefs contribute to the perception of behavioural control.^16^

### Research aim

Utilising an elicitation study within the TPB helps to identify unique beliefs that influence participant behaviour regarding adherence to their treatment. This approach targets fundamental beliefs pivotal for instigating change within a specific context. Thus, the aim of the study was to explore the beliefs affecting ART adherence in newly diagnosed HIV-positive individuals prior to starting ART in Manzini, Eswatini.

## Research methods and design

### Study design

This elicitation study serves as the preliminary stage of a broader research project focused on predicting adherence to HIV care prior to starting ART. Employing an exploratory, qualitative approach, in-depth interviews were conducted with study participants, who were recently diagnosed HIV-positive participants, to understand their beliefs regarding adherence to treatment prior to commencing ART.

### Setting

The data were collected at an HIV clinic situated in Manzini, Eswatini, which is the region with the highest population density (~355 945 residents),^17^ and the highest number of PLHIV (~75 200) and ART recipients (~71 200)^3^ in Eswatini. The local HIV clinic in Manzini, operated by the AIDS Healthcare Foundation, is the largest facility dedicated to HIV care and treatment in the country (Dube N 2017, personal communication).

Eswatini offers a national comprehensive HIV package of care to provide structured treatment to those affected by HIV and AIDS. Upon receiving a positive HIV diagnosis, individuals are promptly enrolled into care and treatment, receiving interventions to promote treatment adherence and regular monitoring.^18^

### Study population and sampling strategy

The target population was individuals living in the Manzini region of Eswatini who had been diagnosed with HIV. Study inclusion criteria were individuals who were aged > 18 years, diagnosed HIV positive on the day of recruitment into the study, enrolled in the ART programme, and initiated onto ART on the day of recruitment. The selection of PLHIV for this qualitative study was purposeful and participants were recruited immediately after a positive HIV test and received no prior counselling about adherence. This timing was chosen due to the pivotal role of counselling in Eswatini’s HIV Care and Treatment Programme, especially post diagnosis.

Nineteen newly diagnosed study participants were interviewed, with the majority (78.9%) being female. This finding is consistent with global research and previous published data from Eswatini, which indicate that women tend to have higher rates of enrolment in ART programmes.^19,20^

### Data collection

From September 2017 to January 2018, qualitative interviews were held with newly diagnosed HIV participants at the local HIV clinic in Manzini, Eswatini. Upon producing a positive HIV test, the healthcare team initially briefed the participants about the study, and those who were interested in participating in the study were subsequently introduced to the researcher, an experienced HIV researcher and psychologist capable of comforting and allaying the fears of a newly informed HIV-positive individual, before enrolment in the HIV care programme. Participants were informed about HIV care and treatment adherence, then asked to share their thoughts on the TPB constructs. The interviews, lasting 15–20 min, used an interview guide made up of open-ended questions adapted from the TPB questionnaire development manual by Francis et al., and adjusted to fit the specific parameters of the research.^21^ During the study, participants were asked to discuss the advantages and disadvantages of engaging in the behaviour of adhering to HIV care and treatment, share their opinions regarding who might be in favour or against this behaviour, and identify factors that could facilitate or impede the adherence behaviour. The interview answers were recorded using a tape recorder.

### Data analysis

Hennink and Kaiser found that saturation can be reached with 9–17 interviews.^22^ They used a base size of nine interviews, with a run length of three interviews (except the last one), and a threshold of ≤ 5% for new information. After nine interviews, 90% saturation was achieved, with only 3.9% new information by the end of interview 19, meeting Guest et al.’s criterion for data saturation in qualitative research.^23^ The data analysis using ATLAS.ti Version 23 followed TPB guidelines proposed by Francis et al.^21^ Thematic analysis was used to analyse interview data. Categories were established initially, then refined into specific themes. The researcher developed themes using participants’ expressions and their own understanding of specific terms. The analysis focused on both frequency and depth of responses during interviews. Data were coded and recoded until no new themes emerged. Themes were then categorised into TPB constructs: behavioural, normative, and control.

Various methods were used to ensure trustworthiness, including pilot interviews, expert evaluation of the interview guide, and translation of data collection tools into Siswati.

### Ethical considerations

This research study was approved by the Human Research Ethics Committee at the University of the Witwatersrand, bearing reference number M210371, and followed ethical guidelines. Study participants received detailed information on the study, gave voluntary written consent to participate in the study, and their answers were assigned a code to de-identify them, to ensure their confidentiality.

## Results

**FIGURE 1:**
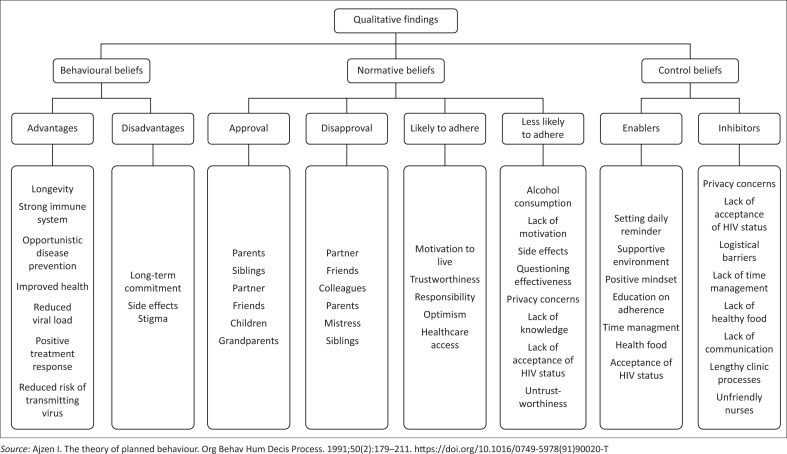
Study findings using the Theory of Planned Behaviour.

Upon examining the belief systems in participants newly diagnosed with HIV, this study revealed a distribution of responses that was dispersed across various categories. Thematic analysis allowed researchers to identify 226 codes, which were then categorised into the three main precursors of the TPB: behavioural, normative, and control beliefs. As illustrated in Figure 1, these precursors are crucial in influencing behavioural intention.

Figure 1 summarises the qualitative findings on HIV treatment adherence, based on behavioural, normative, and control beliefs. The behavioural beliefs list the advantages and disadvantages of adherence. The normative beliefs show the social reactions from different relationships, divided into approval, disapproval, likely to adhere, and less likely to adhere. The control beliefs identify the factors that enable or hinder adherence, categorised into enablers and inhibitors.

The findings suggest that adherence depends on various personal, health, and social factors.

### Behavioural beliefs

Behavioural beliefs reflect how one evaluates a behaviour.^15^

#### Advantages

The findings of the study highlighted the advantages of behavioural beliefs as depicted in Figure 1. Answers to open-ended questions included the following:

A participant underscored longevity as a key advantage, emphasising the potential for increased lifespan, stating:

‘It is for you to live longer than to shorten your life.’ (Participant 9, Female)

This sentiment was reinforced by another participant who emphasised the importance of a strong immune system, stating:

‘[*T*]o boost your immune system.’ (Participant 6, Male)

The prevention of opportunistic diseases was also a significant finding, with a participant asserting:

‘[*S*]ome of the diseases do not attack you frequently like flu.’ (Participant 9, Female)

Health improvement was another notable finding, with one participant sharing:

‘[*R*]egain your health again because if you take them as early as now.’ (Participant 4, Female)

A decrease in viral load was discussed by a study participant who explained how the treatment helped to:

‘[*C*]ontain the virus.’ (Participant 2, Male)

This led to the effect of treatment on their system, as elaborated by a participant:

‘[*W*]here I will be in the know of how the tablets are doing in my system and if there are reactions.’ (Participant 6, Male)

In addition, a study participant acknowledged the lower risk of virus transmission, emphasising the importance of:

‘[*P*]rotecting others from getting the same disease.’ (Participant 3, Female)

These insights provided a valuable understanding of the beliefs associated with these health advantages.

#### Disadvantages

As shown in Figure 1, the findings also highlighted disadvantages alongside the advantages of behavioural beliefs.

Participants recognised the need for long-term commitment, as one participant stated:

‘You have to continue else you die. If you happen to start them, it is a commitment.’ (Participant 17, Female)

Concerns were raised about the side effects of ART, with a participant sharing:

‘I have heard that for a person that is taking them for the first time, the blood gets hot and then you start seeing things, you hallucinate.’ (Participant 18, Female)

The stigma associated with ART was another significant concern. One participant emphasised the issue of discrimination, stating:

‘[*P*]roblem of discrimination, and then the people who take it as a shame to have this disease.’ (Participant 3, Female)

These disadvantages significantly influenced adherence, highlighting the complexity of managing behavioural beliefs in the context of ART.

### Normative beliefs

The findings of the study delved into the realm of normative beliefs as depicted in Figure 1, exploring the impact of social approval and disapproval on the behaviours or actions studied.^15^ The narratives of the participants painted a vivid picture of the social dynamics at play.

#### Approval

Parents emerged as a significant source of approval in the findings. As stated by one participant:

‘They would be happy that I am taking the tablets [*medication*] at the same time all the time.’ (Participant 14, Male)

Another participant also highlighted the role of his mother in his treatment journey, stating:

‘My mom … has already started taking them [*ART*]. She has been telling me that I should go to the clinic.’ (Participant 13, Male)

Partners also played a crucial role in the findings. As emphasised by a participant, transparency in their relationship was important, stating:

‘It is because there are no secrets between us. He has to know about my status.’ (Participant 16, Male)

This sentiment was echoed by another participant, who shared:

‘My husband would be happy because he is already taking them [*ART*] and I can see he is back to health again.’ (Participant 19, Female)

Friends, particularly those also living with HIV, were identified as a supportive group in the findings as shared by a participant, who stated:

‘The people that I am used to talking to, I can say my friends because we normally discuss together as they are also living with HIV/AIDS disease.’ (Participant 13, Male)

Children were seen as potential pillars of support in the findings as expressed by a participant:

‘I also feel that I should also tell my children so that one day when I happen to be sick, they should know what I am suffering from and that they support me.’ (Participant 16, Male)

Lastly, grandparents were also seen as a source of approval in the findings, as shared by a participant:

‘She would be happy that I have chosen the right way that would bring my life back.’ (Participant 4, Male)

These findings underscored the importance of social approval in shaping behaviours and actions, particularly in the context of managing health conditions.

#### Disapproval

However, a concerning theme emerged regarding friends’ disapproval and its impact on adherence to ART. Concurrently, the study revealed the consequential effect of friends’ disapproval on adherence levels.

This delicate social interaction, as depicted in Figure 1, introduced an additional layer of complexity surrounding adherence. The study’s findings emphasised significant concerns among the participants, including fear of contagion, potential distancing, and discrimination. A participant expressed his fear of contagion, stating:

‘Because they will think that they will also catch it from me.’ (Participant 2, Male)

Participants also perceived the potential for distancing as a concern. The participant echoed this sentiment, stating:

‘Because they would think that I am going to infect them and then distance themselves from me. They will be wary of me as though I am no longer a living person.’ (Participant 12, Female)

Participants also grappled with the issue of anticipated discrimination. One participant highlighted this concern, stating:

‘They will have an attitude now that I am positive, and they will discriminate against me.’ (Participant 7, Female)

These insights underscored the complex social dynamics that influence adherence to treatment.

#### Likely to adhere or less likely to adhere

Observational learning is a common strategy employed by individuals when they are uncertain about how to behave in a particular situation. This is especially true when it comes to compliance with HIV care and treatment.

The study noted that individuals who were identified as having a desire to live longer were more likely to adhere to the prescribed guidelines. This was eloquently expressed by one participant, who stated:

‘They are people who love life, who want to live again.’ (Participant 3, Female)

On the other hand, those less likely to adhere to the HIV care plan were often swayed by harmful habits. This was illustrated by a participant, who explained:

‘I think that a person who drinks, who consumes alcohol [*is least likely to adhere*]. That’s what I’m thinking because they would get drunk and forget to take their tablets [*medication*] at the designated time.’ (Participant 19, Female)

These beliefs, shaped by observing others on ART and their outcomes, influenced their adherence motivation, intention, and behaviour. The perceived and anticipated reactions of their close circle of family and friends have potential to significantly impact their adherence to ART.

### Control beliefs

The study’s findings delved into control beliefs as depicted in Figure 1, exploring participants’ perceptions of their ability to adhere to the HIV care and treatment plan. Chang^24^ defined control factors as environmental and psychological factors that influence an individual’s behaviour intention.

#### Enablers

Several enablers were identified. One participant suggested self-reliance and the use of reminders, stating:

‘You can set the alarm if you have one or ask someone to remind you. But ultimately, you have to think for yourself, especially when you are alone.’ (Participant 9, Female)

Another participant emphasised the importance of associating with the right kind of people:

‘[*A*]ssociate myself with the right kind of people.’ (Participant 5, Female)

A participant stressed the need to follow the doctors’ advice:

‘[*S*]tick to what the doctors told you and keep to what they said you should keep up to.’ (Participant 17, Female)

Another participant suggested setting the medication time according to daily routines:

‘[*S*]et the time to what you do on daily basis so that there is no mistake of missing on taking them.’ (Participant 4, Male)

One participant highlighted the importance of a good diet:

‘Have good, healthy food.’ (Participant 16, Male)

Lastly, another participant believed in the power of openness about one’s status to those with whom they live:

‘For me it would work if I tell the people that I am living with that my status is positive so that I am not afraid or shy to take the tablets when it is time to do so.’ (Participant 11, Female)

#### Inhibitors

However, the study also brought to light certain challenges acting as inhibitors of adherence. A participant mentioned logistical barriers, such as distance to the clinic:

‘It could be that I cannot get to the clinic … or that the clinic is far from where I am.’ (Participant 6, Male)

The difficulty of accepting one’s HIV-positive status was another hurdle, as one participant stated:

‘It could be the fact that one has not accepted that they are HIV positive.’ (Participant 1, Female)

Distractions, such as watching a television programme, signalled the poor time management by some participants:

‘[*G*]et delayed because maybe the programme we are watching goes over past nine, that could be the reason that would make it difficult for me to take the tablets.’ (Participant 14, Male)

A participant highlighted the challenge of taking medication on an empty stomach:

‘At times there is no food at home and I know that if I were to take them on an empty stomach, there would be a reaction.’ (Participant 9, Female)

The lengthy hospital processes, as described by a participant, was a deterrent:

‘[*Y*]ou get to the hospital, you will be going from here to there and back here and then right at the end, you get the medication.’ (Participant 7, Female)

Lastly, a participant also mentioned the negative impact of unfriendly nurses:

‘We have another sister [*Nursing*] here that is very cheeky, she shouts and she is just too much.’ (Participant 7, Female)

These insights underscore the complex interplay of control beliefs in influencing adherence to the HIV care and treatment plan.

## Discussion

In this study, an exploration of adherence beliefs among newly diagnosed HIV-positive participants in Manzini, Eswatini, was undertaken using the TPB. The key findings revealed a diverse and widespread array of belief systems, emphasising the importance of considering varied perspectives when addressing adherence challenges.

The behavioural, normative, and control beliefs shed light on factors influencing adherence from the perspective of newly diagnosed HIV-positive participants who have not yet initiated ART, providing a comprehensive understanding of participants’ motivations and barriers to adherence to HIV care.

This study has demonstrated that the factors that could potentially impact adherence to ART in persons who were newly diagnosed with HIV and have not yet initiated treatment were comparable to those that influence adherence in HIV-positive participants who are already taking treatment. Gaining insight into these shared barriers can inform the development of targeted interventions and support strategies that address the specific needs of all HIV-positive individuals, regardless of their treatment status.

### Behavioural beliefs

#### Advantages

The study revealed a spectrum of benefits associated with behavioural beliefs, indicating participants’ perceptions of positive outcomes from adherence to ART. Longevity and enhanced immune function were emphasised, suggesting a desire for a prolonged and healthier life. Participants also highlighted the preventive effects of ART against opportunistic diseases, along with improvements in overall health and a decrease in viral load. These advantages underscored the importance of adherence in managing HIV and preventing transmission. This aligns with the significant benefits of adhering to HIV care and treatment that have been documented in the literature.^8,25^

#### Disadvantages

However, alongside these benefits, participants recognised several challenges associated with adherence to ART. The need for long-term commitment was acknowledged, reflecting the understanding that discontinuation of treatment could lead to adverse consequences. Concerns about potential side effects, including hallucinations, commonly seen in several medications, such as ART,^26^ as well as the discrimination associated with HIV,^5^ were also significant barriers to adherence. These disadvantages highlighted the multifaceted nature of adherence to ART and the complexities individuals face in maintaining such a regimen.

### Normative beliefs

#### Approval

Social support emerged as a crucial factor influencing adherence, particularly from family members, partners, friends, children, and grandparents. Approval from these sources was associated with encouragement and motivation to adhere to treatment. Family members and partners were viewed as pillars of support, providing encouragement and fostering a sense of accountability. These findings underscored the importance of social relations in promoting adherence and improving health outcomes,^27^ whereas participants without social support are more likely to not take their medicine.^28^

#### Disapproval

Conversely, participants expressed concerns about perceived disapproval from friends, stemming from fear of infection with HIV, social distancing, and discrimination. Anticipated negative reactions from friends highlighted the impact on adherence behaviours, emphasising the need for interventions to address social attitudes and misconceptions surrounding HIV and AIDS. A notable theme that emerged pertains to the impact of friends’ disapproval on adherence. Participants expressed concerns about potential contagion fears, social distancing, and discrimination from friends, adding a layer of complexity to the social dynamics influencing adherence.

The study revealed a nuanced interplay of beliefs among participants when discussing discrimination linked to HIV status. This interplay was evident in both normative and behavioural contexts. In terms of normative beliefs, participants expressed anticipation of discrimination from friends and relatives upon disclosing their HIV status. This anticipation stemmed from a perception that societal norms predisposed individuals to develop negative attitudes towards those with HIV.

Conversely, in behavioural beliefs, participants perceived discrimination as a potential consequence of being diagnosed with HIV. This perspective suggests an internalisation of societal attitudes, wherein individuals anticipate experiencing discriminatory behaviour based on their HIV status. These interactions shed light on how participants navigate the complex dynamics of discrimination in their daily lives.

The anticipation of discrimination from social circles underscores the pervasive nature of stigma surrounding HIV, as well as the challenges individuals face in managing societal perceptions of their condition. Moreover, the perception of discrimination as a significant concern highlights the profound impact of societal attitudes on individuals’ psychological well-being and social interactions within their communities.

#### Likely to adhere or less likely to adhere

Those likely to adhere to ART were influenced by a desire for longevity and observed positive outcomes in others on ART. Conversely, those less likely to adhere often succumbed to harmful habits, such as alcohol consumption, which interfered with medication schedules. This confirms the claim that alcohol intake affects HIV progression and survivability by affecting treatment adherence and responsiveness,^29^ which are necessary for lowering HIV levels. These beliefs, shaped by observing others on ART and their outcomes, influenced their adherence motivation, intention, and behaviour.

The normative belief findings underscored the need for targeted interventions that address not only familial support but also the broader social network’s influence on adherence behaviours, as numerous studies have found that HIV stigma reduced the likelihood of HIV treatment, HIV care facility use, ART adherence, and positive treatment outcomes.^30,31,32,33^

### Control beliefs

#### Enablers

Participants identified various factors that facilitated adherence, including self-reliance, reminders, adherence to medical advice, healthy lifestyle choices, and openness about one’s HIV status. These enablers empowered individuals to take control of their health and to adhere effectively to treatment regimens. Supportive environments and access to resources were also identified as key enablers of adherence, highlighting the importance of a holistic approach to HIV care.

#### Inhibitors

Despite these facilitators, participants faced several barriers to adherence, including logistical challenges, difficulties in accepting their HIV-positive status, poor time management, medication side effects, lengthy hospital processes, and negative interactions with healthcare providers. These were consistent with the findings of the study conducted by McNairy et al.^5^ in Eswatini.

These inhibitors underscored the need for comprehensive support systems and interventions to address barriers at individual, interpersonal, and structural levels. Enhancing accessibility, reducing discrimination, and improving participant-provider relationships are essential for overcoming these barriers and promoting adherence.

Strengths of the study findings include a contribution to the breadth of knowledge on ART adherence by offering insights on adherence determinants in participants prior to starting ART, a departure from traditional studies that focused on participants already taking treatment.^34,35,36^ This innovative approach offers a broader understanding of adherence within the ART context. By focusing on the period prior to starting ART, the research addressed a critical gap in the literature, offering insights that can inform early interventions and potentially prevent challenges in adherence before they arise. Early identification of factors influencing adherence allows for targeted and timely interventions, contributing to the UNAIDS 95-95-95 targets.^37^

This proactive approach is especially relevant in Eswatini, where challenges in sustaining optimal adherence have been identified, leading to a lower-than-desired viral suppression rate. Such insights gained from the study can guide the development of personalised counselling strategies, educational initiatives addressing concerns expressed prior to starting ART, and the implementation of support structures anticipating potential barriers to adherence.

In essence, this research contributes to the broader strategy of improving outcomes at each stage of the HIV care continuum. The unique focus on adherence among newly diagnosed participants not yet on ART distinguishes this study as a valuable addition to the field. The findings pave the way for advancing strategies that positively impact the trajectory of HIV treatment outcomes in Eswatini and beyond. As the global community strives for a more personalised and participant-centred approach to HIV care, the insights gleaned from this study offer a promising avenue for advancing interventions that address challenges to ART adherence at their inception.

### Limitations

The TPB offers a structured framework to understand behaviour, which might restrict the assessment of qualitative data and restrict the examination of different interpretations or unforeseen discoveries. Qualitative research needs an adaptable and unrestricted method to data analysis to completely understand the intricacy of behaviour.

## Conclusion and recommendations

This study enhances the understanding of adherence beliefs during the vulnerable post-diagnosis period and prior to starting ART, offering valuable guidance for developing targeted interventions.

The study utilised the TPB as its theoretical framework, and revealed that the factors influencing adherence to ART in individuals newly diagnosed with HIV but not yet taking treatment are comparable to those impacting adherence in HIV-positive participants who are already receiving treatment for HIV.

Additionally, it fills a gap in the existing literature on HIV care and treatment adherence, particularly before the initiation of ART in participants in developing nations. This research lays the foundation for the development of effective strategies in HIV care and treatment.

However, a concerning theme emerged regarding friends’ disapproval impacting adherence. Some participants expressed fear of contagion, potential distancing, and discrimination from their friends. This highlights the need for interventions addressing social influences on adherence, extending beyond familial support.

Further investigation is required to ascertain whether the salient beliefs identified in this study are applicable to a broader population of individuals diagnosed with HIV. The findings of this study can serve as a foundation for future research on the adherence to HIV care and treatment.

In summary, this study not only adds to the theoretical understanding of adherence through the TPB but also offers practical implications for healthcare strategies in Eswatini. The dual focus on familial and social influences on adherence, coupled with the innovative emphasis on identifying adherence determinants prior to starting ART, positions this research as a valuable resource for shaping comprehensive HIV care interventions in Eswatini and similar areas.
